# Letter from Elgin

**Published:** 1881-11

**Authors:** H. Rosencrans

**Affiliations:** Elgin, Ill.


### Domestic Correspondence.

### ARTICLE XII.

THE EDITORS OF THE CHICAGO MEDICAL JOURNAL AND EXAMINER.—The patient here referred to was cured of epilepsy by the *enucleation* of an eye. Mr. K., of Indianola, Texas, for years past had been troubled with epileptic fits. The history given by himself to me was, that about ten years ago he lost the sight of one of his eyes by taking "cold" in it. Since that time he would have spells of his eye inflaming, paining him very much and usually bringing on an epileptic fit. I advised its enucleation. I told him that it was possible and even probable that it might be the means of curring him of his epilepsy. He consented to its removal. On the 9th day of February last I removed it. While under the influence of chloroform and while I was in the act of removing the eye, he was seized with a fit of epilepsy and it took four strong men to hold him on the operating table. I kept up the chloroform, and as soon as his fit left him, lasting about ten minutes, I at once removed the eye. With cooling and antiseptic dressing the socket soon healed. I then, as a constitutional remedy, put him on the use of bromide of potassium. He has had no epeleptic fit since. H. ROSENCRANS, M. D.

ELGIN, ILL., August 18, 1881.

---

THE ninth annual session of the American Public Health Association will be held at Savannah, Ga., Nov. 29 to Dec. 2, inclusive.

